# Experience of Basic Life Support among King Khalid University Health Profession Students, Southwestern Saudi Arabia

**DOI:** 10.3390/ijerph17134822

**Published:** 2020-07-04

**Authors:** Nabil J. Awadalla, Razan S. Al Humayed, Ahmed A. Mahfouz

**Affiliations:** 1 Department of Family and Community Medicine, College of Medicine, King Khalid University, Abha 61421, Saudi Arabia; njgirgis@kku.edu.sa (N.J.A.); R.alhumayed@gmail.com (R.S.A.H.); 2 Department of Community Medicine, College of Medicine, Mansoura University, Mansoura 35516, Egypt; 3 Department of Epidemiology, High Institute of Public Health, Alexandria University, Alexandria 21511, Egypt

**Keywords:** basic life support, experience, health professions students, southwestern Saudi Arabia

## Abstract

Background: Satisfactory experience about basic life support (BLS) is crucial to ensure rapid and efficient delivery of essential life-saving care during emergency situations. Objectives: To assess BLS experience among health profession students at King Khalid University (KKU), Southwestern Saudi Arabia. Methods: A cross-sectional study was conducted on a representative sample of male and female health profession students, during the academic year 2019–2020. A self-reported questionnaire was utilized to collect data about BLS experiences, which included receiving BLS training, reasons for not having BLS training, suggestions to improve BLS training, encountering a situation that required the use of BLS, practicing BLS when needed and reasons for not practicing BLS when needed. Results: Out of 1261 health profession students, 590 received formal BLS training with a prevalence rate of 46.8% (95% CI: 44.0–49.6), and 46.0% of them trained at the university. Important obstacles for non-attendance included busy academic schedule (54.7%) and high cost of the training course (18%). Overall, 84.1% supported integration of BLS training into their college curricula. Almost 26% encountered a situation that required BLS; however, only 32.4% responded. Through multivariate regression, the significant determinant of response was having formal BLS training (aOR = 4.24, 95% CI: 2.38–7.54). The frequent reasons for non-response were lack of adequate BLS knowledge (35.0%), nervousness (22.8%), and that the victim was of opposite sex (9.0%). Conclusion: It is recommended that more emphasis should be given to BLS training among undergraduates of health profession colleges in Southwestern Saudi Arabia. It is recommended that BLS training be integrated into health profession college curricula. Including BLS training as a graduation requirement for health profession students might motivate students to attain BLS training courses.

## 1. Introduction

Cardiovascular diseases (CVDs) are the major cause of worldwide mortality since 1980. In 2015, CVDs were responsible for nearly 30% of all deaths, globally. In Saudi Arabia in 2015, the total number of deaths from CVD amounted to 25,845, and the age standardized death rate for CVD amounted to 231.6 per 100,000. Studies showed that in Saudi Arabia, CVD accounted for more than 40% of all mortalities caused by non-communicable diseases [1]. Studies showed that up to 80% of all cardiac arrests occurred at home [2]. The consequences and prediction of cardiac arrest could be significantly adjusted by the appropriate administration of basic life support (BLS) [3].

Basic life support (BLS) is a collection of crisis activities employed by a person, and it includes several methods, such as cardiopulmonary resuscitation (CPR), shock, and first aid, to support a person’s life until medical assistance arrives or until the patient goes to the hospital. BLS that includes CPR is the first phase after timely detection, and delivers the emergency provision of aeration and circulation in the case of respiratory or cardiac arrest [4]. It includes different procedures such as mouth-to-mouth resuscitation and chest compression, to regularize blood circulation to the brain and important organs. Previous reports stated that timely identification of cardiac arrest, initiation of the emergency medical services (EMS) system, early cardiopulmonary resuscitation, and defibrillation, can influence the distinction between life and death [5]. The timing of cardiopulmonary resuscitation is important and of supreme significance, as asphyxia elicits neuronal impairment within two minutes of cardiac arrest [6].

Numerous reports have examined the knowledge and attitudes of BLS among health profession students in different countries, including Pakistan [7], India [8], Nigeria [9], Egypt [10], and the UK [11]. Similarly, studies in Saudi Arabia assessed BLS knowledge among medical students in Qassim University [12], Jouf University [13], Princess Noura University [14], Jizan University [15], and King Khalid University [16].

Data regarding the experience of health profession students towards BLS in Saudi Arabia are scarce or absent. The aim of the present work was to identify the proportion of health profession students who received BLS training, the barriers to participation, and their BLS experience at King Khalid University, Southwestern Saudi Arabia.

## 2. Materials and Methods

### 2.1. Study Design

A cross-sectional survey study was conducted among male and female health profession students at King Khalid University (KKU), during the 2019–2020 academic year.

### 2.2. Study Setting

KKU is one of the largest public universities in the southwestern area of Saudi Arabia. It is located in the Aseer region. The Aseer region is about 80,000 square kilometers and is populated by more than 2.2 million people. The total number of students in the 2019 academic year was 59,495.

The university includes three health profession colleges, namely, the Colleges of Medicine, Dentistry, and Nursing.

### 2.3. Target Population and Sampling

Students in the Colleges of Medicine, Dentistry, and Nursing were the target population. The academic year in KKU consisted of two levels. Students registered in levels one and two were not related to health profession colleges; indeed, they were enrolled in science colleges and were not included in the present study.

The sample size was estimated utilizing Epi Info software, version 7.2. The total number of students in the three health profession colleges, amounted to 2615. With an anticipated proportion of basic life support (BLS) training among health profession students of 50% and an absolute precision of 3% at 95% confidence, the minimal sample size needed for the survey was calculated to be 1068 students [17]. To account for potential nonresponse, an overall sample size of 1250 students was initially planned for the study.

A multistage stratified cluster sampling technique was employed in choosing the study sample. In each health profession college, students were stratified into the different academic levels. Within each level, a study class, section, or group (cluster) was randomly chosen, using simple random technique sampling. In each selected cluster, all students were included. A total of nearly 1550 students were listed in the chosen clusters. The overall response rate for participation was 81.3% (1261/1550). The most important reasons for non-participation were absence during the survey, incomplete answering of the survey tool, and lack of interest in the study objectives.

### 2.4. Survey Tool

An anonymous, self-reported questionnaire was distributed in person to each participant. Data collection was done by a well-trained group of medical students as part of their training in the community medicine course. The questionnaire collected data regarding the following sections: (a) personal data, such as age and sex; (b) academic characteristics, including level of study, name of college and grade point average (GPA), and (c) self-reported BLS experiences, including having received formal BLS training, reasons for not having had BLS training, suggestions to improve BLS training, encountering a situation that required the use of BLS, practicing BLS when needed and reasons for not practicing BLS when needed.

### 2.5. Data Collection and Ethical Concerns

Before data collection started, permission was granted by the authorities of the assigned colleges to conduct the survey in each setting. The trained students (data collectors) introduced themselves to the students in each selected class, informed them about the research purpose, ensured privacy and data confidentiality, and obtained oral consent. Participation in the survey was entirely voluntary. The average time spent completing the questionnaire was about 7 min.

The survey followed the ethical codes of the pertinent national and institutional committees on human research and the Helsinki Declaration of 1975, as revised in 2008. Ethical approval was obtained from the Ethical Committee of Scientific Research, King Khalid University (ECM#2019-39).

### 2.6. Statistical Analysis

Data were reviewed, refined, and examined using the SPSS software package, version 22. Categorical data were presented as number and percent. Continuous data were presented as mean, standard (SD), and range. Prevalence of having BLS training was presented as point estimate percentage and 95% confidence intervals (CI), and was compared by college and academic level (pre-clinical and clinical). Absence of overlap between the confidence intervals indicated a statistically significant difference. Multivariate logistic regression analysis was operated to explore the independent factors associated with performing BLS when needed. The factors entered in the regression model were gender (male vs. female), academic level (clinical vs. pre-clinical), having BLS training (yes vs. no) and college (Medicine, Dentistry vs. Nursing). Adjusted odds ratios (aOR) and their 95% confidence intervals (CIs) were estimated. *p*-value < 0.05 was regarded as statistically significant.

## 3. Results

### 3.1. Description of the Study Sample

The present study included 1261 health profession students from King Khalid University: 660 from the College of Medicine, 366 from the College of Dentistry and 235 from the College of Nursing. Overall, 711 (56.4%) were males and 550 (43.6%) were females. The College of Nursing is mainly for females. Students were distributed almost equally between pre-clinical (604, 47.9%) and clinical (657, 52.1%) years. The age of the students ranged from 18 to 27 years, with an average of 21.9 ± 1.6 years and a median of 22 years.

### 3.2. Previous Formal Basic Life Support (BLS) Training

Overall, 590 students had received formal basic life support training, giving a prevalence rate of 46.8% (95% CI: 44.0–49.6). Table 1 shows the prevalence percentage and the concomitant 95% confidence intervals of BLS training among the different colleges, by clinical and pre-clinical periods.

Overall, students in clinical years (57.7%, 95% CI: 53.8–61.5) received BLS training more than those in pre-clinical years (34.9%, 95% CI: 31.1–38.9). The difference was significant (as indicated by the lack of overlap between the intervals). The same trend was observed in the Colleges of Medicine, Dentistry, and Nursing.

Overall, among those who had BLS training, almost half of them received BLS at the university during the pre-clinical (46.0%, 95% CI: 39.1–52.9) and clinical years (51.7%, 95% CI: 46.6–56.8). The difference was not statistically significant (as indicated by the overlap between the intervals). Among those who did not receive BLS training, reasons given were busy academic schedule (54.7%) and high cost of the training course (17.9%). Overall, most students (84.1%) suggested that BLS training should be an integral part of their curriculum.

### 3.3. Encountering a Situation that Required the Use of BLS

Almost one out of every four students (330, 26.2%) mentioned that they encountered a situation that required the use of BLS. Figures were different among the different colleges. The highest value was among dentistry (134, 36.6%), followed by nursing (62, 26.4%). The lowest was among medicine (134, 20.3%).

When asked if they did resuscitate the person, almost one third (32.4%, 107) of the students stated that they did. In a multivariate logistic regression analysis (Table 2) the only significant determinant for performing BLS when needed was having formal training in BLS (aOR = 4.245, 95% CI: 2.387–7.549).

Among those who did not interact, the reasons given were lack of knowledge of BLS (35.0%), nervousness (22.8%), victim was of opposite sex (9.0%), and fear of infection via mouth-to-mouth rescue breathing (4.9%).

## 4. Discussion

Satisfactory experience of basic life support (BLS) was crucial for ensuring the rapid and efficient delivery of essential life-saving care during emergency conditions. The focus of the present study was on whether learners had received BLS training, where it occurred, whether they used the BLS training, and barriers to receiving the training. In the present study, up to 46.8% of undergraduate students of the health profession colleges in Southwestern Saudi Arabia had attended BLS training courses. Worldwide, the literature reports varying rates of BLS training among health profession students. In India, one study found that about 19% of medical students received training [8]. Similarly, BLS training was reported to be inadequate among undergraduate students in Pakistan [7], Ethiopia [18], Nigeria [9], and Egypt [19]. On the other hand, in the USA, BLS training was recommended for every healthcare giver since 1966 [20], and most UK medical colleges offer BLS training [21]. In Poland, BLS training was integrated within medical college curricula. In Saudi Arabia, 67.5% of health students in Princess Nourah University previously received BLS training courses [22]. This figure was higher in comparison to our figure. Furthermore, about 91% of the medical and dental interns and 86% of nursing interns had previous BLS training experience in the university hospitals across Saudi Arabia [23]. As BLS training is important to improve knowledge [24] and the self-confidence to practice BLS when required [25,26], there is an urgent need to encourage health profession college undergraduates to attend BLS training courses. The Saudi Council for Health Specialties recommends that students should have formal BLS training before their graduation. Incorporation of BLS training as a condition for graduation of medical students might be necessary to improve BLS training in the pre-graduation stage.

Most students in the current study received BLS training at the clinical stage (57.7%), in comparison to the pre-clinical stage (34.9%). At the clinical stage, students start clinical rotations, which help them to understand the lifesaving importance of BLS and encourage them to gain the adequate knowledge and skills to practice it when needed [25].

In the present study, the most important obstacle to attending BLS training was a busy academic schedule (54.7%). This finding was in agreement with another study carried out in Saudi Arabia, which observed nearly the same result (48%) [24]. Another important obstacle for non-attending reported by about 18% of the students was the high cost of the training course. This result was also in accordance with another study conducted in Saudi Arabia [27]. These results indicate the importance of integrating a BLS training program in a well-disciplined curriculum for all health profession students.

The majority of students (84.1%) in our study suggested that in order to improve BLS training, it should be made an integral part of their curriculum. This result signified a clear positive attitude toward the importance of BLS training to them. In comparison to this finding, a study among health interns among different universities in Saudi Arabia reported that up to 70% of students suggested that BLS training should be included in their college curricula [23]. Various studies recommended including a BLS training course in the college curriculum, for early exposure to BLS experience [22,23,24]. These studies confirmed the importance and benefits of having BLS training at the university. A study showed that BLS training conducted at the university exhibited better results, in comparison to external training [14]. Unfortunately, less than half of the students in the present study had received training at the university.

Our results revealed that about 26% of students encountered a situation that required the use of BLS. In contrast, a study in the UK reported that 61% of students had witnessed cardiac arrest cases (CAs) [28]. In New Zealand, 50% of the newly graduated doctors had witnessed CAs [25], while in Denmark only 3% of the final year students had encountered CAs [26]. This difference could be explained by the difference in the timing of, and time spent, in clinical bed-side training [28]. KKU students in the current cohort started their clinical bed-side teaching late, specifically in the final two to three years of their undergraduate training.

Out of those who encountered a situation that required the use of BLS, only one-third participated in the task of resuscitation. The only determinant for conducting BLS was having BLS training. On the other hand, the most reported barrier to performing BLS, when needed, was a lack of knowledge of BLS. Studies have shown that the willingness to perform BLS might be enhanced by training [27,29]. Lack of knowledge, unsatisfactory training, and the absence of actual practice are barriers to BLS competency [26,28]. Adequate and efficient BLS training for undergraduates is highly recommended.

## 5. Conclusions

In conclusion, it is recommended that more emphasis be placed on BLS training among undergraduates of health profession colleges in Southwestern Saudi Arabia. It is recommended that BLS training be integrated into health profession college curricula, preferably at the pre-clinical stage, and with updated courses presented in the clinical years. Including BLS training as a graduation requirement for health profession students might motivate students to attain BLS training courses.

## Figures and Tables

**Table 1:** Prevalence (%) and 95% CI of basic life support (BLS) training among health profession students at King Khalid University.

BLS Training	College							
	Medicine		Dentistry		Nursing		Total	
	Pre-Clinical	Clinical	Pre-Clinical	Clinical	Pre-Clinical	Clinical	Pre-Clinical	Clinical
Students with BLS training: Prev.% (n/N)	35.8% (103/288)	52.7% (169/372)	42.3% (69/163)	64.0% (130/203)	25.5% (39/153)	64.6% (53/82)	34.9% (211/604)	57.7% (379/657)
95% CI	30.2–41.6	47.5–57.9	34.6–50.3	57.0–70.1	18.8-33.2	53.3–54.9	31.1–38.9	53.8–61.5
Those who had BLS training in university: Prev.% (n/N)	22.3% (23/103)	33.2% (65/169)	76.8% (53/69)	74.6% (97/130)	53.8% (21/39)	64.2% (34/53)	46.0% (97/211)	51.7% (196/379)
95% CI	14.7–31.6	26.6–40.2	65.1–86.1	66.2–81.8	37.2–69.9	49.8–76.9	39.1–52.9	46.6–56.8

n = having BLS; N = number of respondents; and 95% CI: 95% Confidence Interval.

**Table 2:** Multivariate analysis for factors associated with performing BLS upon encountering a situation that required BLS initiation, among health professions students at King Khalid University.

		Performing BLS: aOR (95% CI)
Gender:	Females	Ref 1.025 (0.614–1.712)
	Males	
Academic level:	Pre-clinical	Ref 0.944 (0.573–1.555)
	Clinical	
Taking BLS training:	No	Ref 4.245 (2.387–7.549)
	Yes	
College:	Dentistry and nursing	Ref 0.954 (0.558–1.632)
	Medicine	
